# Beyond Prognosis: A Systematic Review of the Neutrophil-to-Lymphocyte Ratio as a Modifiable Cardiovascular Biomarker

**DOI:** 10.7759/cureus.116277

**Published:** 2026-09-15

**Authors:** Yousof Alemi, Muhammad K Haider, Muhammad S Khan, Muhammad Habeeb Ur Rehman, Patrice D Tate, Karthik Prabakaran, Rabia Saleem, Aeyza Nasir, Ali H Khan

**Affiliations:** 1 Internal Medicine, Peterborough City Hospital, Peterborough, GBR; 2 Internal Medicine, Services Institute of Medical Sciences, Lahore, PAK; 3 Internal Medicine, Foundation University Medical College, Islamabad, PAK; 4 Internal Medicine, Allama Iqbal Medical College, Lahore, PAK; 5 Internal Medicine, Royal Shrewsbury Hospital, Shrewsbury, GBR; 6 Cancer Biology, University of Pennsylvania, Philadelphia, USA; 7 Internal Medicine, King Edward Medical University, Lahore, PAK; 8 Internal Medicine, Nishtar Medical University, Multan, PAK

**Keywords:** atherosclerosis, cardiometabolic therapy, cardiovascular disease, cardiovascular risk, inflammatory biomarkers, myocardial infarction, neutrophil-to-lymphocyte ratio, randomized controlled trials, systemic inflammation

## Abstract

The neutrophil-to-lymphocyte ratio (NLR) is an accessible marker of systemic inflammatory activity associated with adverse cardiovascular outcomes, but whether it is modifiable through intervention and whether its reduction corresponds to cardiovascular benefit remain uncertain. This systematic review evaluated randomized evidence concerning the effects of pharmacological, nutritional, lifestyle, and procedural interventions on NLR among adults with established cardiovascular disease or elevated cardiovascular risk.

MEDLINE via PubMed, Scopus, Web of Science Core Collection, and the Cochrane Central Register of Controlled Trials (CENTRAL) were searched for studies published from January 1, 2021, through July 31, 2026. Eligible studies included randomized controlled trials, randomized crossover trials, and secondary analyses of randomized trials reporting treatment-related changes in NLR. The review followed the Preferred Reporting Items for Systematic Reviews and Meta-Analyses (PRISMA) 2020 guidance. Risk of bias was assessed using the Cochrane Risk of Bias 2 (RoB 2) tool, its crossover adaptation, and the Quality In Prognosis Studies (QUIPS) tool, where appropriate. Twelve studies involving 1,741 participants were included and synthesized narratively. Direct interleukin-6 (IL-6) inhibition produced the clearest dose-dependent reduction in NLR, while glutathione, cardiac rehabilitation, time-restricted eating, curcumin, normobaric hyperoxia, and selected nutritional strategies also demonstrated potentially favorable effects. Findings for sodium-glucose cotransporter 2 inhibitors were inconsistent, with early empagliflozin initiation modulating inflammatory activity in one trial but producing no treatment-specific reduction in NLR during post-infarction recovery in another. Several studies associated lower inflammatory activity with improved ventricular function, global longitudinal strain (GLS), coronary plaque behavior, infarct volume, or neurological severity; however, few formally examined whether NLR independently predicted or mediated these effects. Clinical cardiovascular outcomes were infrequently reported and generally underpowered. Methodological limitations included small sample sizes, heterogeneous measurement schedules, post hoc analyses, multiple biomarker comparisons, and incomplete reporting of NLR outcomes. NLR appears to be a modifiable, context-dependent inflammatory marker, but current randomized evidence does not establish it as a therapeutic target or validated surrogate for cardiovascular benefit. Future trials should prespecify serial NLR assessments and determine whether phenotype-specific changes provide incremental prognostic or treatment monitoring value.

## Introduction and background

Cardiovascular disease remains a leading cause of morbidity and premature mortality despite substantial advances in lipid-lowering therapy, antithrombotic treatment, revascularization, and cardiometabolic risk reduction. Persistent inflammatory activity has emerged as a potential contributor to the residual risk that remains after conventional risk factors have been addressed [1]. Innate immune activation contributes to atherosclerotic plaque formation, myocardial injury, reperfusion responses, ventricular remodeling, and ischemic brain injury, while adaptive immune dysregulation may influence both the intensity and resolution of these processes. However, inflammatory activity is biologically heterogeneous and varies according to disease phenotype, comorbidities, treatment exposure, and the timing of assessment. Identifying an accessible biomarker that reliably captures clinically relevant inflammation could therefore support risk stratification and help clarify the biological effects of cardiovascular interventions [2].

The neutrophil-to-lymphocyte ratio (NLR), calculated from routinely available peripheral blood counts, reflects the balance between innate inflammatory activity and adaptive immune regulation. Neutrophilia may accompany tissue injury, oxidative stress, inflammasome activation, infection, and physiological stress, whereas relative lymphopenia may result from neuroendocrine activation, lymphocyte redistribution, or impaired adaptive immune function. Elevated NLR has consequently been associated with adverse outcomes across coronary artery disease, acute myocardial infarction (AMI), heart failure (HF), stroke, diabetes, obesity, and chronic kidney disease (CKD) [3]. Its low cost and widespread availability make it attractive for clinical investigation, particularly in settings where access to specialized inflammatory biomarkers is limited. Nevertheless, NLR is not specific to cardiovascular inflammation and may be influenced by infection, malignancy, smoking, corticosteroid exposure, renal dysfunction, obesity, and acute physiological stress. An isolated measurement must therefore be interpreted within its clinical context rather than regarded as a direct indicator of a single pathogenic pathway [4].

Much of the existing literature has examined NLR as an observational prognostic marker, while comparatively less attention has been given to whether it can be modified through randomized interventions. This distinction is important because an association with cardiovascular risk does not establish that lowering NLR will improve clinical outcomes. A reduction may indicate direct inhibition of an inflammatory pathway, an indirect metabolic or hemodynamic benefit, or simply the natural resolution of inflammation following acute myocardial injury, percutaneous coronary intervention (PCI), or stroke [5]. Randomized trials of cytokine inhibition, cardiometabolic therapies, nutritional strategies, rehabilitation, dietary modification, and acute procedural interventions provide an opportunity to examine these possibilities. However, the relevant evidence remains dispersed across heterogeneous populations and is frequently reported through secondary or post hoc biomarker analyses. It also remains uncertain whether treatment-related changes in NLR correspond to cardiovascular events, validated surrogate endpoints, or merely parallel changes in intermediate inflammatory markers [1,2].

This systematic review therefore aimed to evaluate the effects of randomized pharmacological, lifestyle, nutritional, and procedural interventions on NLR among adults with established cardiovascular disease or elevated cardiovascular risk. It further examined whether treatment-related changes in NLR were associated with cardiovascular outcomes or clinically relevant surrogate endpoints, including ventricular function, cardiac remodeling, coronary plaque burden, infarct volume, and reperfusion measures. By distinguishing biological responsiveness from prognostic validity and therapeutic mediation, this review sought to clarify whether NLR is best understood as a contextual marker of inflammatory activity, a pharmacodynamic indicator of treatment response, or a potential target for future cardiovascular research.

## Review

Materials and methods

Study Design and Reporting Framework

This systematic review evaluated how randomized pharmacological, nutritional, lifestyle, and procedural interventions influence the neutrophil-to-lymphocyte ratio (NLR) in adults with established cardiovascular disease or elevated cardiovascular risk. The review was conducted and reported in accordance with the Preferred Reporting Items for Systematic Reviews and Meta-Analyses (PRISMA) 2020 statement [6]. The protocol was not prospectively registered because registration was not initiated before the study selection process; retrospective registration was not pursued, as it would not constitute genuine prospective registration. Nevertheless, the research question, eligibility criteria, study selection process, outcomes, and synthesis strategy were defined before final study inclusion, and no substantive methodological amendments were made during the review. Owing to substantial clinical and methodological heterogeneity in the study populations, interventions, NLR measurement schedules, and reported outcomes, the evidence was synthesized narratively rather than through statistical pooling.

Research Question and Population, Intervention, Comparator, and Outcome (PICO) Framework

The review question was structured according to the Population, Intervention, Comparator, and Outcome (PICO) framework. The population comprised adults aged 18 years or older with established cardiovascular or cerebrovascular disease, including coronary artery disease, acute myocardial infarction, unstable ischemic heart disease, atherosclerosis, cardiac structural abnormalities, or ischemic stroke. Adults with conditions conferring elevated cardiovascular risk, such as type 2 diabetes mellitus (T2DM), obesity, chronic kidney disease, systemic inflammation, or left ventricular hypertrophy, were also eligible. Interventions included pharmacological, anti-inflammatory, cardiometabolic, nutritional, lifestyle, rehabilitation, and acute procedural strategies evaluated within a randomized design. Comparators included placebo, usual care, no intervention, an active treatment, delayed treatment initiation, or the control period of a randomized crossover trial. The primary outcome was the between-group or within-participant change in NLR. Secondary outcomes included cardiovascular mortality, major adverse cardiovascular events (MACE), myocardial infarction, stroke, heart failure hospitalization, and clinically relevant cardiovascular or cerebrovascular measures, including ventricular structure and function, global longitudinal strain (GLS), coronary plaque burden, infarct volume, neurological severity, coronary reperfusion, and inflammatory or oxidative stress biomarkers.

Information Sources and Search Strategy

MEDLINE via PubMed, Scopus, Web of Science Core Collection, and the Cochrane Central Register of Controlled Trials (CENTRAL) were searched for studies published from January 1, 2021, through July 31, 2026. This period was selected to capture contemporary randomized evidence concerning modification of the neutrophil-to-lymphocyte ratio (NLR) in cardiovascular and cardiometabolic populations. The final database search was conducted on July 31, 2026. The strategy combined controlled vocabulary, where available, with free-text terms relating to NLR, cardiovascular or cardiometabolic conditions, and randomized interventions. Synonyms within each concept were combined using “OR,” and the concept groups were linked using “AND.” Truncation, subject headings, and field restrictions were adapted to the indexing and search structure of each database. Table 1 presents the databases, representative search terms, and Boolean approach. Reference lists of included articles and relevant reviews were also examined for potentially eligible randomized studies or secondary trial analyses. No geographical restrictions were applied.

**Table 1:** Summary of the electronic literature search strategy NLR: neutrophil-to-lymphocyte ratio

Database	Search terms	Boolean operators and database-specific adaptation
MEDLINE via PubMed	(“neutrophil-to-lymphocyte ratio” OR “neutrophil lymphocyte ratio” OR “neutrophil/lymphocyte ratio” OR NLR); (“cardiovascular disease” OR “cardiovascular risk” OR “coronary artery disease” OR atherosclerosis OR “myocardial infarction” OR “acute coronary syndrome” OR “percutaneous coronary intervention” OR “heart failure” OR “ventricular remodelling” OR stroke OR diabetes OR obesity OR “chronic kidney disease”); (randomized OR randomised OR “randomized controlled trial” OR “clinical trial” OR placebo OR “controlled trial” OR crossover OR intervention)	Synonyms within each term group were combined using OR, and the three groups were linked using AND. Medical Subject Headings and field restrictions were applied where appropriate.
Scopus	Same core NLR, cardiovascular or cardiometabolic, and interventional search terms	OR within term groups and AND between groups; syntax, truncation, and title, abstract, and keyword fields were adapted for Scopus.
Web of Science Core Collection	Same core NLR, cardiovascular or cardiometabolic, and interventional search terms	OR within term groups and AND between groups; syntax, truncation, and topic-field searching were adapted for Web of Science.
Cochrane Central Register of Controlled Trials (CENTRAL)	Same core NLR, cardiovascular or cardiometabolic, and interventional search terms	OR within term groups and AND between groups; syntax and subject headings were adapted for CENTRAL.

Eligibility Criteria

Eligibility was determined according to study design, population, NLR reporting, and availability of relevant data, as summarized in Table 2.

**Table 2:** Study inclusion and exclusion criteria NLR: neutrophil-to-lymphocyte ratio

Criterion	Inclusion criteria	Exclusion criteria
Study design	Randomized controlled trials, randomized crossover trials, and secondary or post hoc analyses preserving the randomized comparison	Observational, nonrandomized, single-arm, case-control, cross-sectional, preclinical, animal, or in vitro studies
Population and clinical relevance	Participants with established cardiovascular disease or a recognized cardiovascular or cardiometabolic risk condition	Populations without established cardiovascular disease or a recognized cardiovascular risk condition
NLR reporting	NLR reported before and after an intervention or sufficient comparative data available; NLR could be a primary, secondary, exploratory, or post hoc endpoint	Only total leukocyte, neutrophil, or lymphocyte counts reported; NLR findings not separately extractable; or “NLR” used for an unrelated term
Publication and data availability	Full reports providing sufficient NLR and intervention-related data	Reviews, meta-analyses, editorials, protocols, case reports, letters without original data, conference abstracts with insufficient data, and duplicate reports without additional relevant findings

Study Selection

All retrieved records were combined, and duplicate citations were removed electronically and confirmed manually. Two reviewers independently screened titles and abstracts against the predefined eligibility criteria. Articles considered potentially relevant by either reviewer underwent full-text assessment. The same reviewers independently evaluated the full texts and recorded the principal reason for each exclusion. Disagreements at either stage were resolved through discussion and consensus; when consensus could not be reached, a third reviewer was consulted. The study selection process was documented using a PRISMA 2020 flow diagram.

Data Extraction

Two reviewers independently extracted data using a standardized data collection form. Extracted information included the first author, publication year, trial design, clinical setting, sample size, participant characteristics, cardiovascular condition or risk phenotype, intervention, comparator, treatment duration, follow-up period, and timing of blood collection. NLR data included baseline values, follow-up values, absolute or percentage changes, between-group differences, confidence intervals, and probability values, where reported. Information was also collected on cardiovascular events, ventricular structure and function, coronary plaque characteristics, infarct volume, neurological outcomes, reperfusion measures, CRP, cytokines, oxidative stress indices, and other relevant inflammatory markers. For secondary and post hoc analyses, the relationship to the parent randomized trial and whether the original randomized comparison was preserved were recorded. Disagreements in extracted data were resolved by re-examination of the source article and reviewer consensus.

Risk-of-Bias Assessment

Risk of bias was assessed independently by two reviewers using an outcome-specific approach. Randomized parallel-group trials and randomized secondary analyses that retained the original treatment comparison were evaluated with the Cochrane Risk of Bias 2 tool [7]. This assessment considered the randomization process, deviations from intended interventions, missing outcome data, outcome measurement, and selection of the reported result. The randomized crossover study was evaluated using the RoB 2 version adapted for crossover trials, including consideration of period and carryover effects. Prognostic or responder analyses based on post-randomization classifications were additionally evaluated using the Quality In Prognosis Studies (QUIPS) tool [8], particularly when the principal finding concerned the relationship between baseline NLR, treatment response, or subsequent clinical outcomes rather than the randomized intervention effect itself. Disagreements were resolved through consensus or consultation with a third reviewer.

Data Synthesis

Studies were grouped according to the principal mechanism of intervention, including direct inflammatory modulation, cardiometabolic therapy, oxidative or nutritional intervention, lifestyle modification, and acute procedural treatment. Findings were interpreted according to the direction, magnitude, timing, and statistical significance of NLR change, with separate consideration of whether the change differed from the comparator. Associations between NLR and hard cardiovascular outcomes, validated surrogate endpoints, and intermediate biological markers were distinguished to avoid equating biomarker responsiveness with clinical benefit. Meta-analysis was not undertaken because of substantial heterogeneity in participant phenotype, intervention mechanism, comparator, follow-up duration, NLR reporting, and outcome definition. Particular attention was given to whether observed NLR changes could be separated from spontaneous recovery following acute myocardial infarction, PCI, or stroke, and whether any study formally demonstrated that NLR independently predicted or mediated cardiovascular benefit.

Results

Study Selection Process

As shown in Figure 1, the database search identified 741 records, of which 32 duplicates were removed before screening. The titles and abstracts of the remaining 709 records were screened, resulting in the exclusion of 532 records. Of the 177 reports sought for retrieval, 20 could not be obtained, leaving 157 reports for full-text assessment. A further 145 reports were excluded because of nonrandomized study design, non-original or preclinical evidence, absent NLR reporting, ineligible populations, unrelated NLR terminology, or duplication. Ultimately, 12 studies met the eligibility criteria and were included in the systematic review.

**Figure 1:**
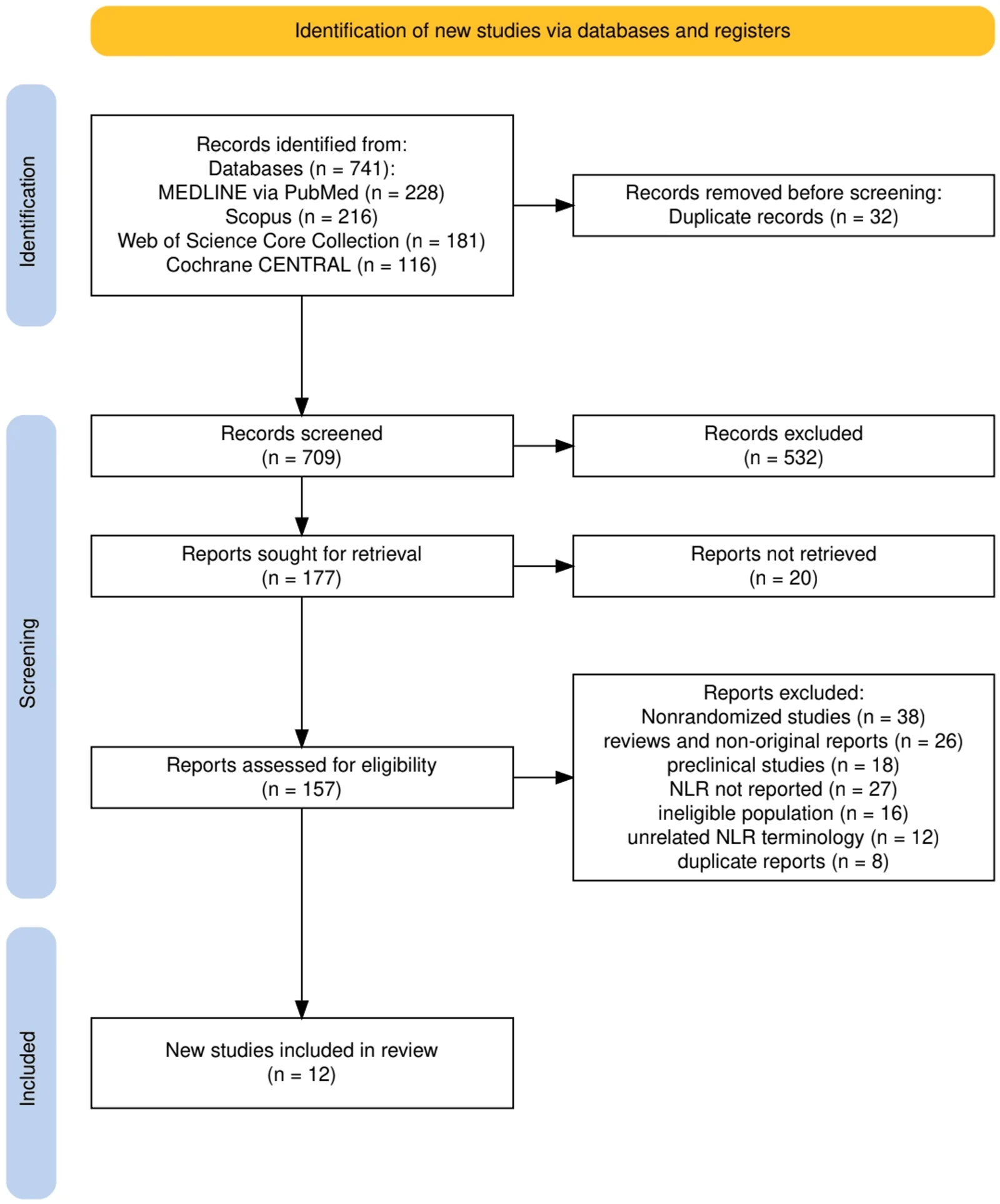
PRISMA flow diagram of the study identification, screening, eligibility assessment, and inclusion process NLR: neutrophil-to-lymphocyte ratio, PRISMA: Preferred Reporting Items for Systematic Reviews and Meta-Analyses

Characteristics of the Selected Studies

Twelve randomized trials or secondary analyses of randomized trials, comprising 1,741 participants, were included (Table 3). Sample sizes ranged from 19 to 374 participants, and follow-up extended from 24 hours after an acute intervention to 5 years. Populations included patients with myocardial infarction, coronary artery disease, PCI, ischemic stroke, left ventricular hypertrophy, diabetes, obesity, and CKD-associated cardiovascular risk. Interventions encompassed anti-inflammatory and cardiometabolic therapies, nutritional supplementation, dietary modification, cardiac rehabilitation, hyperoxia, and procedural pharmacotherapy. NLR was generally evaluated as a secondary or exploratory biomarker, with considerable variation in measurement timing and outcome reporting.

**Table 3:** Characteristics and principal findings of randomized studies evaluating intervention-related changes in the neutrophil-to-lymphocyte ratio AMI: acute myocardial infarction, ATP: adenosine triphosphate, AUC: area under the curve, CI: confidence interval, CKD: chronic kidney disease, CORDIOPREV: Coronary Diet Intervention with Olive Oil and Cardiovascular Prevention, CRP: C-reactive protein, DAPA-LVH: Does Dapagliflozin Regress Left Ventricular Hypertrophy in Patients With Type 2 Diabetes?, DHA: docosahexaenoic acid, EMMY: Impact of Empagliflozin on Cardiac Function and Biomarkers of Heart Failure in Patients With Acute Myocardial Infarction, EPA: eicosapentaenoic acid, FPG: fasting plasma glucose, GLS: global longitudinal strain, HbA1c: glycated hemoglobin, HF: heart failure, HOMA-IR: homeostatic model assessment of insulin resistance, HR: hazard ratio, hs-CRP: high-sensitivity C-reactive protein, IL: interleukin, IQR: interquartile range, IV: intravenous, LV: left ventricular, MACE: major adverse cardiovascular events, MI: myocardial infarction, NIHSS: National Institutes of Health Stroke Scale, NLR: neutrophil-to-lymphocyte ratio, NLRP3: NOD-like receptor family pyrin domain-containing 3, NOX2: nicotinamide adenine dinucleotide phosphate oxidase 2, OPENS: Normobaric Hyperoxia Combined With Reperfusion for Acute Ischaemic Stroke, PCI: percutaneous coronary intervention, PLR: platelet-to-lymphocyte ratio, RCT: randomized controlled trial, RESCUE: Trial to Evaluate Reduction in Inflammation in Patients With Advanced Chronic Renal Disease Utilizing Antibody-Mediated Interleukin-6 Inhibition, SC: subcutaneous, STEMI: ST-segment elevation myocardial infarction, T2DM: type 2 diabetes mellitus, TIMI: Thrombolysis in Myocardial Infarction, TNF: tumor necrosis factor

Study	Design and population	Intervention versus comparator	NLR assessment	Effect on NLR	Clinical/surrogate outcome and landmark finding	Key statistical results
Adamstein et al. (2023) (RESCUE) [9]	Post hoc analysis of a multicenter, double-blind phase 2 RCT; 264 adults with moderate-to-severe CKD and hs-CRP ≥ 2 mg/L; 48% had atherosclerosis	Ziltivekimab 7.5, 15, or 30 mg SC every four weeks versus placebo; 24 weeks	Percentage change from baseline to week 12	Dose-dependent reduction in NLR, driven partly by reduced absolute neutrophil count	Demonstrated direct pharmacological modulation of NLR through IL-6 inhibition; cardiovascular outcomes were not evaluated	Median NLR change: placebo, +1.56%; 7.5 mg, -13.5%; 15 mg, -14.3%; and 30 mg, -22.4%; treatment differences versus placebo were -14.6%, -15.3%, and -23.6%, respectively; all P≤0.004
Boughanem et al. (2025) (CORDIOPREV) [10]	Secondary analysis of an RCT; 183 patients with coronary heart disease and newly diagnosed, untreated T2DM	Mediterranean diet versus low-fat diet for five years	Baseline and five years; NLR and other neutrophil-derived indices examined	NLR decreased more among patients achieving diabetes remission; a direct randomized dietary effect on NLR was not clearly established	Lower baseline neutrophil burden identified patients more likely to achieve T2DM remission with a Mediterranean diet	NLR change in remission responders versus nonresponders: P=0.035; neutrophil count at five years: P=0.006; lowest versus highest neutrophil tertile with Mediterranean diet: adjusted HR 4.23 (95% CI: 1.53-11.69); prediction model AUC: 0.783
Cliff et al. (2026) [11]	Randomized timing trial; 66 patients with T2DM following AMI	Empagliflozin initiated at discharge versus delayed for 90 days; follow-up 180 days	Days 0, 30, 90, 120, and 180	Early empagliflozin reduced NLR by day 90, with the effect sustained after continued treatment; no comparable early reduction occurred without treatment	Early treatment attenuated macrophage NLRP3 activation and inflammatory and senescence pathways; cardiovascular events were not assessed	NLR reduction by day 90: P=0.006; CRP: P=0.0047; IL-1β expression decreased by approximately 70% with early treatment versus 32% with delayed treatment; ATP: P=0.005; TNF secretion: P=0.02
Yaikwawong et al. (2026) [12]	Double-blind, placebo-controlled RCT; 114 participants with obesity and T2DM included in the primary analysis	Curcumin 1500 mg/day versus placebo for 12 months	Baseline and 12 months	Curcumin produced a modest but statistically significant reduction in NLR compared with placebo	Curcumin improved inflammatory, antioxidant, and glycemic profiles; cardiovascular outcomes were not evaluated	At 12 months, median NLR was 1.61 (IQR: 1.23-1.92) with curcumin versus 1.82 (1.31-2.34) with placebo, P=0.029; effect size r=0.27 (95% CI: 0.12-0.41); median change was -0.12 (IQR: 0.53) versus +0.13 (0.70), respectively, P=0.029
Niu et al. (2026) (OPENS) [13]	Secondary analysis of an RCT; 86 patients with acute ischemic stroke and anterior circulation large vessel occlusion undergoing thrombectomy	Normobaric hyperoxia, consisting of 100% oxygen at 10 L/min for four hours, plus thrombectomy versus thrombectomy alone	Baseline and 24 hours after randomization	Normobaric hyperoxia significantly attenuated the early post-thrombectomy increase in NLR	24-hour NLR was associated with neurological severity, change in NIHSS score, and infarct volume	At 24 hours, median NLR was 9.12 (IQR: 6.26-12.05) versus 11.00 (9.28-14.88); adjusted difference: -2.64 (95% CI: -5.27 to -0.02), P=0.042; 24-hour NLR correlated with 24-hour NIHSS (r=0.591), change in NIHSS (r=0.424), and infarct volume (r=0.513); all P<0.001
Dihoum et al. (2024) (DAPA-LVH) [14]	Secondary biomarker analysis of an RCT; 60 patients with T2DM and LV hypertrophy without symptomatic HF	Dapagliflozin 10 mg/day versus placebo for 12 months	Baseline and 12 months	No significant between-group reduction in NLR	Reduction in NLR correlated modestly with improved GLS, but not with LV mass	Correlation between NLR change and GLS improvement: r=0.311; P-value not reported; CRP mean difference: -1.96 (95% CI: -3.68 to -0.24), P=0.026
Los et al. (2026) [15]	Randomized, open-label, blinded-endpoint crossover trial; 19 patients with previous MI	Two weeks of time-restricted eating from 08:00 to 14:00 versus usual diet; washout of at least six weeks	Before and after each two-week intervention period	Time-restricted eating significantly reduced NLR compared with the control diet	Time-restricted eating reduced neutrophil counts and low-grade inflammation and produced anti-inflammatory monocyte-transcriptomic changes; cardiovascular events were not assessed	NLR changed from 2.1±0.8 to 1.9±0.6 with time-restricted eating and from 1.9±0.6 to 2.1±0.7 with the control diet; the between-intervention difference in change was -0.4 (95% CI:-0.8 to -0.1); nominal P=0.016 and adjusted P=0.040
Benedikt et al. (2023) (EMMY) [16]	Post hoc biomarker analysis of a multicenter, double-blind RCT; 374 patients following AMI and PCI	Empagliflozin 10 mg/day, initiated within 72 hours of PCI, versus placebo for 26 weeks	Baseline through week 26	NLR declined substantially over time in both groups, but empagliflozin produced no additional reduction compared with placebo	The cardiac benefits observed in EMMY were unlikely to be mediated by attenuation of the measured systemic inflammatory response	Overall NLR reduction, 10.22±0.50%, P<0.001; hs-CRP, -57.4%; IL-6, -35.1%; and neutrophils, -26.1%; no significant treatment-group difference was observed
Welty et al. (2023) [17]	Secondary analysis of an RCT; 240 patients with stable coronary artery disease	EPA plus DHA 3.36 g/day versus no supplementation for 30 months	Baseline and follow-up; analyses stratified by blood pressure and plaque response status	Significant NLR reduction occurred only among normotensive participants receiving EPA plus DHA	Plaque progression was associated with substantially more cardiac events; direct mediation by NLR was not demonstrated	Cardiac events: plaque progressors 22.3% versus regressors 5.0%, P<0.001; risk ratio: 2.89 (95% CI: 1.1-8.0), P=0.034; triglyceride reduction correlated with plaque regression: r=0.35, P=0.034
Awada et al. (2024) [18]	RCT; 115 patients with unstable ischemic heart disease following successful PCI	12-week cardiac rehabilitation (n=77) versus usual-care control (n=38)	Baseline and 12 weeks	NLR was significantly lower following cardiac rehabilitation	Rehabilitation attenuated several inflammatory biomarkers; the trial was not powered for cardiovascular events	NLR: 2.17 (IQR: 1.42-2.43) versus 2.26 (2.07-2.60), P=0.016; hs-CRP: P<0.001; PLR: P=0.027
Tanzilli et al. (2021) [19]	Placebo-controlled RCT; 100 patients with STEMI undergoing primary PCI	IV glutathione 2500 mg immediately before PCI and again at 24, 48, and 72 hours versus placebo	At admission and five days after reperfusion	Glutathione significantly reduced NLR following reperfusion compared with placebo	Attenuation of the inflammatory response was associated with improved subsequent LV size and function	NLR results were presented graphically; treatment-by-time interaction P<0.0001, with between-group and within-glutathione comparisons P<0.0005; the broader inflammatory response was associated with LV outcomes (r=0.78, P<0.005)
Wu et al. (2025) [20]	RCT; 120 patients with AMI undergoing PCI	Bivalirudin versus unfractionated heparin	Before PCI and on postoperative day 7	NLR reportedly decreased by approximately 25%, but no clear between-group NLR advantage was demonstrated	Bivalirudin reduced bleeding and improved final coronary flow, whereas 30-day MACE was identical between groups	TIMI grade 3 flow: 85% versus 70%; MACE: 5% versus 5%, P>0.05; bleeding rates were lower with bivalirudin; a significant between-group NLR difference was not established

Effects of Individual Interventions on NLR

The effects of pharmacological interventions varied according to their mechanism and clinical context. Ziltivekimab produced a dose-dependent reduction in NLR, with median changes ranging from -13.5% to -22.4% across the three doses, compared with an increase of 1.56% with placebo [9]. Early initiation of empagliflozin after acute myocardial infarction reduced NLR by day 90 [11]. In contrast, the EMMY analysis found that NLR declined during post-infarction recovery in both treatment groups, without an additional effect from empagliflozin [16]. Dapagliflozin also produced no significant between-group reduction in NLR, although declining NLR was modestly correlated with improvement in global longitudinal strain [14].

Among nutritional interventions, curcumin produced a modest but statistically significant reduction in NLR. At 12 months, the median NLR was 1.61 in the curcumin group and 1.82 in the placebo group (P=0.029) [12]. In the CORDIOPREV analysis, NLR decreased more among participants who achieved diabetes remission, although a direct randomized effect of the Mediterranean diet on NLR was not clearly demonstrated [10]. Eicosapentaenoic acid (EPA) plus docosahexaenoic acid (DHA) reduced NLR only among normotensive participants, indicating possible variation according to blood pressure phenotype [17]. Two weeks of time-restricted eating reduced NLR relative to the control diet, with a between-intervention difference in change of -0.4 (95% confidence interval (CI): -0.8 to -0.1; adjusted P=0.040) [15].

Cardiac rehabilitation was associated with a lower NLR after 12 weeks compared with usual care (2.17 versus 2.26; P=0.016) [18]. In acute care settings, normobaric hyperoxia attenuated the early increase in NLR following thrombectomy for ischemic stroke. At 24 hours, the median NLR was 9.12 with hyperoxia plus thrombectomy and 11.00 with thrombectomy alone (adjusted difference: -2.64; 95% CI: -5.27 to -0.02; P=0.042) [13]. Glutathione administered before and for three days after primary percutaneous coronary intervention significantly reduced post-reperfusion NLR compared with placebo (treatment-by-time interaction P<0.0001) [19]. Bivalirudin was associated with an overall decline in NLR after percutaneous coronary intervention, but a clear advantage over unfractionated heparin was not demonstrated [20].

Quality Assessment

The risk-of-bias assessment is presented in Table 4 and Table 5. Eleven intervention effect estimates were evaluated using RoB 2 or its crossover trial adaptation. Ten were judged to have some concerns, and one was judged to have a high risk of bias. Concerns most commonly involved missing biomarker data, lack of prospectively documented NLR-specific analyses, and the availability of multiple eligible measurements, time points, or subgroup analyses. Objective laboratory measurement of NLR resulted in a low risk of measurement bias across studies. Small sample size and absence of participant blinding were not treated as automatic sources of bias; instead, their potential effects were evaluated within the relevant RoB 2 domains. Prognostic and associational analyses were assessed separately using QUIPS.

**Table 4:** RoB 2 assessment of intervention effects on NLR CORDIOPREV: Coronary Diet Intervention with Olive Oil and Cardiovascular Prevention, DAPA-LVH: Does Dapagliflozin Regress Left Ventricular Hypertrophy in Patients With Type 2 Diabetes?, EMMY: Impact of Empagliflozin on Cardiac Function and Biomarkers of Heart Failure in Patients With Acute Myocardial Infarction, MI: myocardial infarction, NLR: neutrophil-to-lymphocyte ratio, OPENS: Normobaric Hyperoxia Combined With Reperfusion for Acute Ischaemic Stroke, PCI: percutaneous coronary intervention, QUIPS: Quality In Prognosis Studies, RCT: randomized controlled trial, RESCUE: Trial to Evaluate Reduction in Inflammation in Patients With Advanced Chronic Renal Disease Utilizing Antibody-Mediated Interleukin-6 Inhibition, RoB 2: Risk of Bias 2, T2DM: type 2 diabetes mellitus

Study	Randomization process	Deviations from intended interventions	Missing outcome data	Measurement of NLR	Selection of reported result	Overall judgement	Supporting basis
Adamstein et al. (2023) (RESCUE) [9]	Low	Low	Some concerns	Low	Some concerns	Some concerns	The parent trial used random allocation, placebo control, and blinding. NLR was objectively measured, but week 12 data were unavailable for some participants following COVID-19-related interruption, and the NLR analysis was not clearly specified in the prospective analysis plan.
Cliff et al. (2026) [11]	Some concerns	Some concerns	Low	Low	Some concerns	Some concerns	Participants were randomized to early or delayed empagliflozin, but sequence generation and allocation concealment were not fully described. The open-label timing design could influence adherence or co-interventions, although NLR measurement was objective. NLR was one of several inflammatory outcomes assessed at multiple time points without a clearly available prospective NLR analysis plan.
Yaikwawong et al. (2026) [12]	Low	Low	Some concerns	Low	Some concerns	Some concerns	Computer-generated block randomization, allocation concealment, and double blinding were described. Substantial attrition occurred, although intention-to-treat and sensitivity analyses supported the primary result. The prospective documentation of the specific NLR analysis and available analytical choices was not fully clear.
Niu et al. (2026) (OPENS) [13]	Low	Some concerns	Low	Low	Some concerns	Some concerns	The analysis originated from a randomized trial and used an objective laboratory outcome. Blinding to hyperoxia was difficult, although this was unlikely to influence NLR measurement directly. The NLR analysis was secondary and included several inflammatory markers and time points without a clearly documented prospective NLR-specific analysis plan.
Dihoum et al. (2024) (DAPA-LVH) [14]	Low	Low	Some concerns	Low	Some concerns	Some concerns	The parent trial was randomized and placebo-controlled, and NLR was objectively measured. The biomarker analysis used an available-sample subset of the parent trial, and NLR was evaluated alongside several inflammatory markers and exploratory correlations without a clearly documented prospective NLR analysis plan.
Los et al. (2026) [15]	Low	Some concerns	Low	Low	Some concerns	Some concerns	The randomized crossover design incorporated a washout period and accounted for intervention period and participant effects. The open-label dietary intervention could affect adherence or behavior, although NLR measurement was objective. An explicit carryover effect assessment and prospectively specified NLR analysis were not clearly reported.
Benedikt et al. (2023) (EMMY) [16]	Low	Low	Some concerns	Low	Some concerns	Some concerns	The parent trial used randomization, placebo control, and double blinding. The post hoc biomarker analysis included a subset with available measurements, and NLR was assessed among several inflammatory outcomes without a prospectively documented NLR-specific analysis plan.
Welty et al. (2023) [17]	Low	Some concerns	Some concerns	Low	Some concerns	Some concerns	Treatment allocation originated from a randomized trial, and NLR measurement was objective. However, the analysis depended on available follow-up measurements and reported effects within blood pressure and plaque response subgroups. The prespecification of the NLR subgroup analyses was unclear.
Awada et al. (2024) [18]	Some concerns	Some concerns	Some concerns	Low	Some concerns	Some concerns	Randomization was reported, but sequence generation and allocation concealment were insufficiently detailed. Participant blinding was impractical, which could influence adherence or additional care, although NLR measurement was objective. Attrition handling and prospective specification of the NLR analysis were not fully described.
Tanzilli et al. (2021) [19]	Low	Low	Low	Low	Some concerns	Some concerns	Computer-generated randomization, identical placebo infusion, and blinding of participants, investigators, laboratory personnel, and outcome assessors were described. Missing data were limited, and NLR was objectively measured. Some concern remained because NLR was a secondary biomarker and its numerical results were presented graphically without a clearly available prespecified NLR analysis plan.
Wu et al. (2025) [20]	High	Some concerns	Some concerns	Low	High	High	The report stated that participants were randomly allocated but did not adequately describe sequence generation or allocation concealment. Blinding, protocol deviations, and missing data handling were insufficiently reported. Treatment-specific NLR estimates and statistical comparisons were incomplete or internally unclear, creating a substantial risk of selective outcome reporting.

**Table 5:** QUIPS assessment of prognostic and associational analyses QUIPS: Quality In Prognosis Studies, NLR: neutrophil-to-lymphocyte ratio, T2DM: type 2 diabetes mellitus

Study	Study participation	Study attrition	Prognostic factor measurement	Outcome measurement	Study confounding	Statistical analysis and reporting	Overall judgement	Supporting basis
Boughanem et al. (2025) (CORDIOPREV) [10]	Moderate	Moderate	Low	Low	Moderate	Moderate	Moderate risk	The analysis included a selected subgroup of participants with newly diagnosed T2DM and classified them according to remission achieved after randomization. Neutrophil indices and remission were objectively measured, and adjusted analyses were performed; however, subgroup selection, attrition over five years, post-randomization responder classification, and possible residual confounding limited causal interpretation.
Welty et al. (2023) [17]	Moderate	Moderate	Low	Low	High	High	High risk	NLR, plaque measures, and cardiac events were measured using defined methods. However, analyses based on post-randomization plaque progression or regression were vulnerable to confounding and selection effects. Multiple comparisons across treatment, blood pressure, and plaque response subgroups increased the risk of selective reporting.

Discussion

Principal Findings and Overall Interpretation

This systematic review indicates that the neutrophil-to-lymphocyte ratio (NLR) may be modified by a diverse range of pharmacological, lifestyle, nutritional, and procedural interventions in adults with established cardiovascular disease or elevated cardiovascular risk. Reductions were observed following direct cytokine inhibition, antioxidant therapy, cardiac rehabilitation, dietary modification, and selected acute care interventions, although their magnitude and consistency varied considerably across clinical settings.

Importantly, several interventions produced no treatment-specific reduction in NLR despite improving other inflammatory or cardiovascular measures, suggesting that NLR does not uniformly capture the biological effects of cardiovascular therapies. Moreover, most trials evaluated NLR as a secondary or exploratory biomarker and were neither designed nor adequately powered to establish its relationship with cardiovascular mortality or major adverse cardiovascular events. The available evidence therefore supports NLR primarily as a modifiable indicator of systemic inflammatory activity rather than as a validated surrogate endpoint for cardiovascular benefit.

NLR as a Marker, Mediator, or Therapeutic Target

The biological interpretation of NLR requires recognition that it integrates several overlapping processes rather than reflecting a single inflammatory pathway. Neutrophilia may accompany innate immune activation, oxidative stress, tissue injury, inflammasome signaling, and acute physiological stress, whereas relative lymphopenia may reflect neuroendocrine stress or altered adaptive immune regulation.

In a secondary analysis of the Trial to Evaluate Reduction in Inflammation in Patients With Advanced Chronic Renal Disease Utilizing Antibody-Mediated Interleukin-6 Inhibition (RESCUE), Adamstein et al. [9] demonstrated a dose-dependent reduction in NLR with interleukin-6 ligand inhibition, supporting its potential role as a pharmacodynamic marker when an intervention directly engages a defined inflammatory pathway. Similarly, Tanzilli et al. [19] reported that glutathione reduced NLR alongside oxidative and inflammatory biomarkers following primary percutaneous coronary intervention (PCI). By contrast, Benedikt et al. [16] found that NLR declined substantially during recovery from acute myocardial infarction but was not additionally reduced by empagliflozin, illustrating how the natural resolution of post-infarction inflammation may resemble a treatment response.

Collectively, these findings suggest that NLR may reflect disease activity, spontaneous recovery, or pharmacological response, depending on the clinical context. Its reduction should therefore not be assumed to represent interruption of a causal cardiovascular pathway unless it precedes, independently predicts, or mediates a clinically meaningful treatment effect.

Intervention-Specific Patterns of NLR Modification

The intervention-specific patterns presented in the Results suggest that NLR responsiveness may depend on how directly a treatment engages the inflammatory processes represented by the ratio. Direct cytokine inhibition produced the clearest treatment-specific signal, whereas cardiometabolic, nutritional, lifestyle, and acute care interventions produced more variable effects [9-20]. This heterogeneity may reflect differences in biological specificity, treatment duration, baseline inflammatory burden, population phenotype, and measurement timing. Consequently, the absence of an NLR reduction should not be interpreted as evidence that an intervention lacks cardiovascular or anti-inflammatory benefit, while an observed reduction should not by itself be considered evidence of clinical effectiveness.

Timing and Clinical Context as Determinants of NLR Response

The interpretation of NLR change is inseparable from the temporal and clinical context in which it is measured. Following acute myocardial infarction, PCI, or ischemic stroke, neutrophil recruitment, lymphocyte redistribution, oxidative stress, reperfusion injury, and neurohormonal activation may produce rapid fluctuations in NLR, followed by a spontaneous decline as tissue injury and systemic stress resolve. Against this dynamic background, an observed reduction may appear treatment-related unless it is compared with an appropriately timed control trajectory.

The studies by Cliff et al. [11] and Benedikt et al. [16] illustrate this distinction. Early empagliflozin initiation was associated with earlier modulation of inflammatory activity than delayed treatment, whereas substantial declines in NLR during post-infarction recovery occurred in both the empagliflozin and placebo groups in the EMMY analysis. Acute reductions observed with glutathione and normobaric hyperoxia should likewise be interpreted within the rapidly changing biology of reperfusion and tissue injury [13,19].

In stable coronary disease, diabetes, and chronic ventricular remodeling, inflammatory activity is generally less dynamic, and treatment-related changes may emerge more gradually or remain modest. Changes in NLR are therefore most informative when evaluated against the expected untreated trajectory using standardized measurement intervals appropriate to the disease stage and intervention mechanism.

Relationship Between NLR Change and Cardiovascular Benefit

Although several studies linked NLR or broader inflammatory changes with cardiovascular surrogates, the available evidence does not establish NLR reduction as a mediator of clinical benefit. Reported associations involved left ventricular structure and function, global longitudinal strain, coronary plaque behavior, infarct volume, and neurological severity [13,14,17,19]. However, these findings were generally based on composite inflammatory responses, exploratory correlations, or post-randomization subgroup analyses. Few studies formally examined whether NLR independently predicted outcomes or mediated the randomized treatment effect.

This distinction is clinically important because cardiovascular death, recurrent myocardial infarction, and stroke represent clinical outcomes, whereas ventricular function, plaque burden, and infarct volume are surrogate endpoints. CRP, cytokines, and oxidative stress markers remain intermediate biological measures. For example, the improvements in bleeding and coronary flow reported with bivalirudin did not translate into a difference in 30-day major adverse cardiovascular events [20].

The current evidence therefore supports NLR as a biologically responsive marker more strongly than as a validated cardiovascular surrogate. Establishing clinical utility will require trials demonstrating that treatment-related changes in NLR predict subsequent events, improve risk discrimination, or explain a measurable proportion of therapeutic benefit independently of established biomarkers.

Patient Phenotype and Sources of Heterogeneity

Variation in NLR responses across the included trials may reflect meaningful biological heterogeneity rather than inconsistency alone. Baseline inflammatory burden, diabetes, obesity, chronic kidney disease (CKD), blood pressure status, disease acuity, and established atherosclerosis can each influence circulating neutrophil and lymphocyte counts and the capacity of an intervention to modify them.

The findings of Welty et al. [17] suggest that hemodynamic phenotype may influence inflammatory responsiveness because the reduction in NLR with EPA plus DHA was confined to normotensive participants. Similarly, Boughanem et al. [10] identified relationships between neutrophil burden, dietary response, and diabetes remission, although baseline NLR did not independently predict remission. The dose-dependent response to ziltivekimab occurred in a population characterized by CKD and elevated high-sensitivity C-reactive protein (hs-CRP), which may limit its generalizability to patients with lower inflammatory activity [9].

Interpretation is further complicated by non-cardiovascular determinants of NLR, including infection, corticosteroid exposure, smoking, malignancy, obesity, renal dysfunction, and physiological stress. These factors may alter NLR independently of cardiovascular disease or treatment response. Consequently, a universal threshold may obscure clinically important differences among patient groups. Phenotype-specific reference ranges and within-patient trajectories may therefore provide more meaningful information than isolated measurements interpreted without adequate clinical context.

Methodological Strengths, Limitations, and Interpretation of the Evidence

Restricting this review to randomized trials and their secondary analyses strengthens the causal interpretation of intervention effects compared with evidence derived solely from observational studies. Nevertheless, the randomized design of a parent trial does not eliminate bias within post hoc biomarker analyses, particularly when participants are subsequently classified according to treatment response, blood pressure status, or clinical outcome. NLR was rarely a prespecified primary endpoint, and several studies were limited by small samples, short follow-up periods, or incomplete reporting of absolute and percentage changes. Measurement schedules ranged from several hours after an acute intervention to five years after dietary modification, limiting direct comparison across studies. Multiple inflammatory biomarkers and assessment time points were also frequently examined, increasing the possibility of chance findings when correction procedures or prespecified analysis plans were not clearly reported. Cardiovascular mortality and major adverse cardiovascular events were generally absent or insufficiently powered, while relationships with surrogate endpoints were often exploratory and potentially susceptible to residual confounding. Differences in disease phenotype, intervention mechanism, and background therapy further reduced clinical comparability. Most reports also lacked standardized adjustment for infection, smoking, corticosteroid use, malignancy, renal dysfunction, and other determinants of leukocyte counts. Publication bias and selective reporting remain possible because neutral NLR findings may be less likely to receive detailed presentation. Collectively, these limitations support the use of structured narrative synthesis and preclude firm conclusions regarding the comparative efficacy of individual interventions for reducing NLR.

Clinical Implications and Priorities for Future Trials

NLR is inexpensive, widely available, and easily derived from routine complete blood counts, making it an attractive candidate for monitoring inflammatory activity in cardiovascular practice. However, the present evidence does not support using NLR reduction alone to select therapy, assess treatment success, or infer cardiovascular protection. Future trials should prespecify NLR as an exploratory or secondary endpoint, define standardized sampling intervals, and obtain repeated measurements capable of capturing early, intermediate, and sustained trajectories. Analyses should distinguish treatment-related changes from the natural inflammatory course of acute illness and report both absolute and relative changes with appropriate between-group estimates. Investigators should also determine whether NLR provides incremental prognostic information beyond CRP, troponin, natriuretic peptides, renal function, and established clinical risk models. Phenotype-stratified analyses could establish whether baseline inflammatory burden, diabetes, CKD, obesity, hypertension, or acute tissue injury identifies populations in whom NLR is most informative. Most importantly, adequately powered trials should evaluate associations with cardiovascular death, recurrent myocardial infarction, stroke, heart failure hospitalization, and validated structural or functional endpoints. Formal mediation analyses would help determine whether NLR lies within a therapeutic pathway or merely changes alongside it. Until such evidence becomes available, NLR is best regarded as a promising contextual biomarker rather than an established treatment target or validated surrogate for cardiovascular benefit.

## Conclusions

Randomized evidence indicates that NLR can be modified by pharmacological, lifestyle, nutritional, and procedural interventions, although its response varies according to treatment mechanism, patient phenotype, disease stage, and measurement timing. Direct inflammatory inhibition provided the clearest evidence of a treatment-specific effect, whereas responses to cardiometabolic and lifestyle interventions were less consistent and often difficult to distinguish from natural recovery. Importantly, evidence that lowering NLR independently predicts or mediates cardiovascular benefit remains limited. The central take-home message is that NLR should currently be interpreted as a dynamic, context-dependent marker of inflammatory activity rather than a universal therapeutic target or validated surrogate endpoint. Its clinical value will depend not simply on whether NLR decreases, but on whether standardized, phenotype-specific changes provide incremental prognostic information and reliably correspond to meaningful improvements in cardiovascular outcomes.
